# Complement Activation is More Pronounced in the Kidneys of Critically Ill Patients With COVID-19 Than in Those With Bacterial Sepsis

**DOI:** 10.1016/j.ekir.2025.04.027

**Published:** 2025-04-21

**Authors:** Firas F. Alkaff, Grietje G. Talen, Marius C. van den Heuvel, Meint Volbeda, Matijs van Meurs, Jill Moser, Mohamed R. Daha, Jacob van den Born, Stefan P. Berger

**Affiliations:** 1 Division of Nephrology, Department of Internal Medicine, University of Groningen, University Medical Center Groningen, Groningen, The Netherlands; 2 Division of Pharmacology and Therapy, Department of Anatomy, Histology, and Pharmacology, Faculty of Medicine, Universitas Airlangga, Surabaya, Indonesia; 3 Department of Pathology and Medical Biology, Pathology Section, University of Groningen, University Medical Center Groningen, Groningen, The Netherlands; 4 Department of Critical Care, University of Groningen, University Medical Center Groningen, Groningen, The Netherlands; 5 Department of Pathology and Medical Biology, Medical Biology Section, Laboratory for Endothelial Biomedicine and Vascular Drug Targeting Research, University of Groningen, University Medical Center Groningen, Groningen, The Netherlands; 6 Department of Nephrology, Leiden University Medical Center, Leiden, Netherlands

**Keywords:** acute kidney injury, complement, COVID-19, kidney, sepsis

## Abstract

**Introduction:**

Previous studies have shown that the complement system plays an important role in COVID-19 acute kidney injury (AKI). However, studies evaluating the activation pathways *in vivo* are scarce and have shown contradictory findings. It has also been suggested that COVID-19 AKI has a pathophysiology similar to that of bacterial sepsis AKI. Nonetheless, no study has compared the complement activation between these 2 types of AKI.

**Methods:**

This study used postmortem kidney tissue from 22 patients with COVID-19 and 22 patients with bacterial sepsis. Control kidney tissues were obtained from 12 patients who underwent total nephrectomy. Immunohistochemical staining was performed for complement factor properdin and complement activation products C4d, C3d, and C5b-9. Glomerular and tubulointerstitial complement deposition was quantitatively analyzed using ImageScope.

**Results:**

Peritubular capillary thrombi were found in 82% of the biopsies in the COVID-19 group but were absent in the bacterial sepsis group. Both C3d and properdin positivity in the tubulointerstitial area were significantly higher in COVID-19 than in bacterial sepsis (*P* = 0.034 and *P* = 0.001, respectively) and in the control group (*P* = 0.034 and *P* < 0.001, respectively) and were predominantly found in the peritubular capillaries. In contrast, no difference was found in tubulointerstitial C4d and C5b-9 positivity between the COVID-19 and the bacterial sepsis groups.

**Conclusions:**

There was marked tubulointerstitial complement deposition in the kidneys of patients with COVID-19, particularly in the peritubular capillaries, with activation via the alternative pathway. Thus, alternative complement pathway inhibition might be a possible treatment option for patients with COVID-19 AKI.

## Graphical abstract

**Figure:**
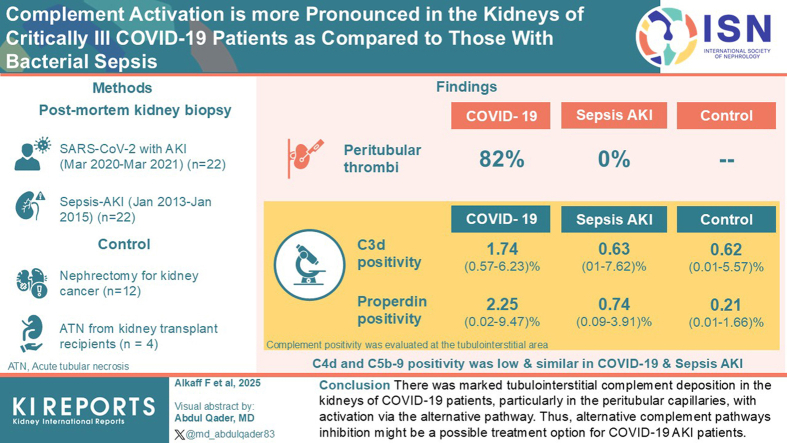

COVID-19 is an infectious disease caused by the SARS-CoV-2 virus. It was first identified in December 2019 and caused a global pandemic. Although pneumonia and respiratory failure are the predominant clinical features associated with COVID-19, SARS-CoV-2 infection can also directly or indirectly cause deleterious effects on many organ systems, including the kidney.^1^

AKI is a common complication in patients with COVID-19. A previous systematic review and meta-analysis reported that the pooled prevalence of AKI among hospitalized patients with COVID-19 was 28%, with a higher prevalence observed in critically ill patients with COVID-19 treated in the intensive care unit.^2^ Similar to AKI from other causes, COVID-19 AKI is associated with a higher risk of mortality.^3^^,^^4^

The pathophysiology of COVID-19 AKI is thought to be multifactorial and involves endothelial injury, coagulopathy, and dysregulation of immune responses, including the complement system.^5^ To better understand the pathogenesis of COVID-19, tissue-based pathological studies are required.^6^^,^^7^ Previous studies have reported that the terminal complement complex C5b-9 is deposited in most kidney tissues of patients with COVID-19, primarily in the tubular basement membrane or peritubular capillaries.^8^, ^9^, ^10^, ^11^, ^12^ This suggests that the complement system plays a central role in the pathogenesis of COVID-19 AKI.

The complement system can be activated via 3 different pathways: classical, lectin, and alternative pathways. The classical pathway is activated by C1q binding to an antigen-antibody complex. The lectin pathway is activated when mannose-binding lectins, ficolins, or collectins encounter foreign or altered carbohydrate or acetylated structures. Activation of either the classical or lectin pathway leads to cleavage of C4.^13^ The alternative pathway is constantly activated via a “tick-over” mechanism or via properdin binding to certain microbial surfaces and activated, apoptotic, or necrotic cells.^13^^,^^14^ The complement system in the kidney is extensively reviewed elsewhere.^15^ Regardless of the activation pathway, complement activation leads to cleavage of C3, resulting in the formation of C5b-9.^13^ Identifying the activation pathways involved in disease pathogenesis can help determine the potential of complement-targeted therapy in critically ill patients with COVID-19 with AKI.

Despite the high number of histopathological studies of COVID-19 kidney biopsies,^16^ only 4 studies evaluated the complement activation pathway.^9^, ^10^, ^11^, ^12^ However, these studies have reported contradictory findings. Two studies concluded that the complement system was activated via the classical and alternative pathways.^9^^,^^12^ Another study indicated that complement activation occurred via the lectin and alternative pathways,^10^ and another study reported that complement activation occurred exclusively via the lectin pathway (Supplementary Table S1).^11^ Furthermore, no previous studies have evaluated properdin deposition, although properdin is known to activate the complement system via an alternative pathway.^14^ It has been suggested that COVID-19 AKI has a pathophysiology similar to that of bacterial sepsis.^17^, ^18^, ^19^ However, the differences and similarities in complement activation between these 2 diseases in critically ill patients have not been studied in detail.

Considering that thrombotic complications and coagulopathy frequently occur in COVID-19 and are involved in the pathophysiology of COVID-19 AKI,^5^^,^^20^ and that the complement and coagulation cascades are highly interconnected,^21^ we hypothesized that complement is involved in COVID-19-related kidney damage. We hypothesized that the activation pathway is mainly via the alternative pathway, similar to what has been shown in AKI from other causes.^22^^,^^23^ We aimed to evaluate this hypothesis by comparing complement activation in postmortem kidney biopsies of severely ill patients with COVID-19 and those with bacterial sepsis relative to that in control kidney tissues.

## Methods

### Ethical Clearance

This study was approved by the Medical Ethics Review Committee of the University Medical Center Groningen (METc 2011/372 and Research Register Number: 201900431). Permission and written informed consent were obtained at the final family meetings. For the kidney transplant recipients’ biopsies, in the extension of the ongoing TransplantLines Biobank and Cohort study (NCT03272841), the Medical Ethics Review Committee of the University Medical Center Groningen gave an exemption from requiring written informed consent for studies that use clinical data and leftover material (including the kidney biopsy) of all kidney transplant recipients in University Medical Center Groningen (METc 2014/077). The study was conducted in accordance with the World Medical Association Declaration of Helsinki and the Declaration of Istanbul.

### Kidney Biopsies

#### Bacterial Sepsis

Postmortem kidney biopsies were collected from adult patients who died with sepsis AKI between January 2013 and January 2015 (*n* = 27), as described in detail elsewhere.^24^ However, 5 biopsies were deemed inadequate (explained in the next section), leaving 22 biopsies for analysis. All patients were classified as having septic shock according to the International Sepsis Definition.^25^ AKI severity stage was categorized according to the applicable AKI criteria at that time, that is, RIFLE (Risk, Injury, Failure, Loss, and End-stage kidney disease) criteria.^26^ The severity of critical illness was defined upon admission to the intensive care unit using the Simplified Acute Physiology Score II.^27^

#### COVID-19

Postmortem kidney biopsies were collected from adult patients with SARS-CoV-2 infection who died between March 2020 and March 2021 (*n* = 22). The diagnosis of COVID-19 was confirmed by reverse-transcriptase polymerase chain reaction of oropharyngeal and nasopharyngeal swabs. SARS-CoV-2 RNA in the kidney was evaluated by real-time reverse-transcriptase polymerase chain reaction on E-gen and N-gen, as previously described.^28^ The AKI severity stage was categorized according to the latest Kidney Disease: Improving Global Outcomes criteria.^29^ The severity of critical illness was evaluated using the Simplified Acute Physiology Score II.^27^

#### Control

Control kidney biopsies were collected from a healthy part of kidneys that underwent a complete nephrectomy for kidney cancer (*n* = 12). An experienced pathologist (MCvdH) assessed the control biopsies and confirmed that they looked like normal kidney tissue.

#### Acute Tubular Necrosis (ATN) Control

Because complement C3d deposition is upregulated in the tubular basement membranes of kidneys with ATN,^30^ we included additional ATN controls with similar degree of ATN extensiveness as COVID-19. For this, we included kidney biopsies from kidney transplant recipients who had undergone an indication biopsy after transplantation and had ATN without infection or rejection (*n* = 4).

### Tissue Sampling and Histopathological Evaluation

Kidney biopsies were harvested from patients with bacterial sepsis and those with COVID-19 under ultrasound guidance after the biopsy device (Angiotech, 14 Ga × 20 cm, Tru Core2 Biopsy Instrument, Gainesville, FL) was introduced through a small (5–7 mm) skin incision. All biopsies were taken as quickly as possible after death at the bedside in our intensive care unit and therefore considered “warm,” thereby eliminating unwanted tissue necrosis and autolysis that might influence the analysis. Control biopsy was performed within 30 minutes after the nephrectomy procedure. Kidney biopsy tissues were immediately fixed in 10% formalin fixative for 24–48 hours and subsequently processed and embedded in paraffin.^24^^,^^28^

For histopathological evaluation, deparaffinized sections were stained with hematoxylin and eosin, periodic acid–Schiff, and Martius Scarlet Blue. All sections were evaluated and manually scored by the same experienced kidney pathologist (MCvdH) following routine pathological procedures; therefore, the evaluation could not be blinded. Glomeruli were evaluated for glomerular sclerosis, presence of thrombi, and an increase in the mesangial matrix. The tubules were evaluated for ATN, tubulitis, interstitial inflammation, and interstitial fibrosis and tubular atrophy. The microvasculature was evaluated for the presence of thrombi and peritubular capillaritis. Detailed scoring methods have been described previously^24^^,^^28^ and are presented in Supplementary Table S2.

### Complement Staining and Evaluation

Paraffin-embedded kidney tissues were cut into 3 μm-thick sections, deparaffinized in xylene, and rehydrated in graded ethanol. The staining of complement factors properdin, C3d, and C5b-9 was performed by immunohistochemistry with the following primary antibodies: polyclonal rabbit antihuman properdin (Nephrology Lab, Leiden University Medical Center, Leiden, the Netherlands), polyclonal rabbit antihuman C3d (A0063, DAKO, Carpinteria, CA), and monoclonal mouse antihuman C5b-9 (A239, Quidel, San Diego, CA).^31^^,^^32^ Kidney biopsies from deceased donors were used as positive controls.^32^^,^^33^ Additional positive control from a patient with primary membranous nephropathy was also included because biopsy from primary membranous nephropathy has been known to be positive for the evaluated complement factors.^34^ For the negative control, the primary antibody was replaced with phosphate-buffered saline (17-512Q; Lonza, Wijchen, the Netherlands).^33^ The detailed staining protocol is presented in Supplementary Table S3. To avoid day-to-day variation and allow for comparison between biopsies, all staining per complement factor were performed on the same day with the same reagents, antibody dilutions, and incubation times.

C4d staining was performed using a Benchmark Ultra automated IHC/ISH slide staining system (Ventana Medical Systems, Roche Diagnostics, Almere, the Netherlands). This system is used for diagnostic purposes in the University Medical Center Groningen (Groningen, the Netherlands) pathology department. The staining system and the ready-to-use antibodies were validated by the manufacturer and verified by our pathology department.^32^

Slides stained for complement factors were digitalized using a Hamamatsu slide scanner (Hamamatsu Photonics, Hamamatsu, Japan) and analyzed using Aperio ImageScope software (Leica Biosystems, Nussloch, Germany). Quantitative scoring was performed by calculating the percentage of positivity in (up to) 10 tubulointerstitial areas (without glomeruli and large blood vessels) in the cortex (111,459.25 μm^2^ per area, equivalent to 0.33 × 0.33 mm) and (up to) 15 glomeruli using the build-in positive pixel count v9 algorithm. If the biopsy had < 4 tubulointerstitial areas and/or 4 glomeruli, it was excluded. Therefore, we excluded 5 biopsies from the bacterial sepsis group, leaving 22 biopsies for the analyses.

The positive pixel count v9 algorithm categorizes pixel positivity into the following 4 categories: negative, weakly positive, positive, and strongly positive pixels. The percentage of staining positivity, defined as the sum of positive and strong positive pixels multiplied by 100 and divided by the total number of pixels in the selected area,^35^ was used to quantitatively represent complement deposition in the tissue. Because each complement staining differs, we adjusted the thresholds of several input parameters (i.e., hue width, hue value, and color saturation threshold) in the algorithm. After adjustment, the percentage positivity of the glomeruli and peritubular areas in the negative control group was 0.00%. After the threshold of the input parameter had been set, all biopsies of 1 complement factor staining were evaluated using the same settings. The percentage of positivity used for the analyses was derived from the median value of all the selected tubulointerstitial areas (or glomeruli). An example of the percentage of positive quantification is shown in Supplementary Figure S1.

### Statistical Analyses

Statistical analyses were performed using IBM SPSS Statistics for Windows version 25.0. (IBM Corp., Armonk, NY). For descriptive statistics, continuous variables are presented as medians (lower range–upper range). Categorical variables are expressed as numbers (valid percentages). Mann Whitney-U test and Fisher exact test were used to compare the clinical characteristics, histopathological findings, and complement staining positivity between groups. Statistical significance was defined as a *P*-value < 0.05. However, because multiple tests were performed to compare the difference in the percentage of complement staining positivity between groups (control vs. sepsis, control vs. COVID-19, and sepsis vs. COVID-19), the *P*-value was corrected using the false discovery rate method.^36^ The correction was performed separately for each complement staining.

## Results

### Patient Characteristics

Patient characteristics are presented in Table 1. The age and prevalence of the female sex were similar in patients with bacterial sepsis and COVID-19. However, body mass index was higher among patients with COVID-19 (29.8 [23.9–38.3] vs. 27 [20.8–32.7] kg/m^2^, *P* = 0.001). One-third of the patients in the bacterial sepsis group had normal body mass index (18.5–24.9 kg/m^2^), whereas only 1 patient in the COVID-19 group had normal body mass index. None of the patients in the bacterial sepsis group and only 1 patient in the COVID-19 group was diagnosed with prior kidney disease.

**Table 1:** Baseline characteristics of the study population

Variables	Control *n* = 12	ATN Control *n* = 4	Sepsis *n* = 22	COVID-19 *n* =22	*P*-value^a^
Age, yrs	62 [20–79]	50 [42–69]	66 [40–85]	71 [49–78]	0.6
Female sex, *n* (%)	7 (58)	1 (25)	8 (36)	9 (41)	1.0
BMI, kg/m^2^	n/a	n/a	27 [20.8–32.7]	29.8 [23.9–38.3]	0.001
Comorbidities, *n* (%)					-
Kidney disease	0 (0)	4 (100)	0 (0)	1 (5)	
Hypertension	3 (25)	0 (0)	8 (36)	6 (27)	
Vascular disease	1 (8)	0 (0)	5 (23)	1 (5)	
Type 2 diabetes	1 (8)	1 (25)	2 (9)	3 (14)	
COPD or asthma	4 (33)	0 (0)	5 (23)	5 (23)	
Malignancy	4 (33)	0 (0)	3 (14)	2 (9)	
Autoimmune	2 (17)	0 (0)	6 (27)	2 (9)	
RIFLE stage					-
Risk			0 (0)		
Injury			8 (36)		
Failure			14 (64)		
Loss of function			0 (0)		
End-stage			0 (0)		
KDIGO AKI					-
0				7 (32)	
1				0 (0)	
2				0 (0)	
3				15 (68)	
SAPS II score	n/a	n/a	66 [46–99]	46 [30–71]	<0.001
Duration in ICU, d	n/a	n/a	3 [1–12]	13 [2–36]	<0.001
KRT during hospitalization, *n* (%)	n/a	n/a	11 (50)	8 (36)	0.5
Creatinine level at admission, μmol/l	n/a	n/a	139 [82–419]	98 [48–341]	0.051
Highest creatinine level during hospitalization, μmol/l	n/a	n/a	175 [90–475]	234 [50–513]	0.6
Biopsy time, min	n/a	n/a	35 [25–150]	20 [6–50]	0.016

BMI, body mass index; COPD, chronic obstructive pulmonary disease; ICU, intensive care unit; KDIGO AKI, Kidney Disease: Improving Global Outcomes Acute kidney injury; KRT, kidney replacement therapy; RIFLE, (Risk, Injury, Failure, Loss, and End-stage kidney disease); SAPS II, The Simplified Acute Physiology Score II.

a *P*-value between bacterial sepsis and the COVID-19 group. Continuous variables were presented as median [lower range-upper range], and categorical variables were presented as frequency (valid percentage). The Mann-Whitney U test was used for continuous variables, and the Fisher exact test was used for categorical variables.

All patients in the bacterial sepsis and COVID-19 groups were mechanically ventilated. Compared with the COVID-19 group, the sepsis group had more severe illness upon admission as evidenced by the higher Simplified Acute Physiology Score II score (66 [46–99] vs. 46 [30–71], *P* < 0.001) and a shorter stay in the intensive care unit until death (3 [1–12] d vs. 13 [2–36] d, *P* < 0.001). Fifteen of 22 patients (68%) in the COVID-19 group and all patients in the sepsis group were clinically diagnosed with AKI. However, there was no significant difference in the number of patients requiring kidney replacement therapy during hospitalization (50% vs. 36%, *P* = 0.5) (Table 1).

### Histopathological Findings

SARS-CoV-2 RNA was not detected in the kidneys of patients with COVID-19 in our cohort. In line with our earlier paper,^28^ thrombi in peritubular capillaries were found in the majority (82%) of patients with COVID-19 but were absent in all bacterial sepsis patients. Next, ATN was more extensively distributed in the biopsies of patients with COVID-19 than in those of bacterial sepsis patients (2 [2–3] vs. 1 [0–3], *P* < 0.001), despite the similar ATN morphology stage. In the glomerular area, the only histopathological findings were glomerular thrombi, and it was only observed in 1 patient in each group (Table 2). Autolysis was not observed in biopsies included in this cohort. As for the ATN control group, all had interstitial inflammation, but none had thrombi in the peritubular capillaries. Regarding the ATN extensiveness, it was either 2 or 3, similar to the COVID-19 group. The detailed histopathological scores for each patient are presented in Supplementary Table S4.

**Table 2:** Histopathology findings in the kidney biopsy of patients with bacterial sepsis and COVID-19

Histopathology findings in the kidney	Bacterial sepsis *n* = 22	COVID *n* =22	*P*-value
Glomerulus			
Glomerulitis, *n* (%)	0 (0)	0 (0)	1.0
Thrombi glomerular, *n* (%)	1 (4)	1 (4)	1.0
Increase mesangial matrix score ≥ 1, *n* (%)	0 (0)	0 (0)	1.0
Peritubular			
Acute tubular necrosis morphology stage, *n* (%)			0.6
0	3 (14)	0	
1	3 (14)	8 (36)	
2	15 (68)	14 (64)	
3	1 (4)	0	
Acute tubular necrosis extensiveness, *n* (%)			< 0.001
0	3 (14)	0	
1	15 (68)	0	
2	2 (9)	13 (59)	
3	2 (9)	9 (41)	
4	0	0	
Tubulitis score ≥ 1, *n* (%)	0 (0)	2 (9)	0.5
Interstitial inflammation score ≥ 1, *n* (%)	2 (9)	3 (14)	1.0
Interstitial fibrosis/tubular atrophy score ≥ 1, *n* (%)	7 (32)	5 (23)	0.7
Peritubular capillaritis score ≥ 1, *n* (%)	0 (0)	1 (4)	1.0
Thrombi peritubular capillaries, *n* (%)	0 (0)	18 (82)	< 0.001

### Complement Deposition and the Activation Pathway

**Figure 1:**
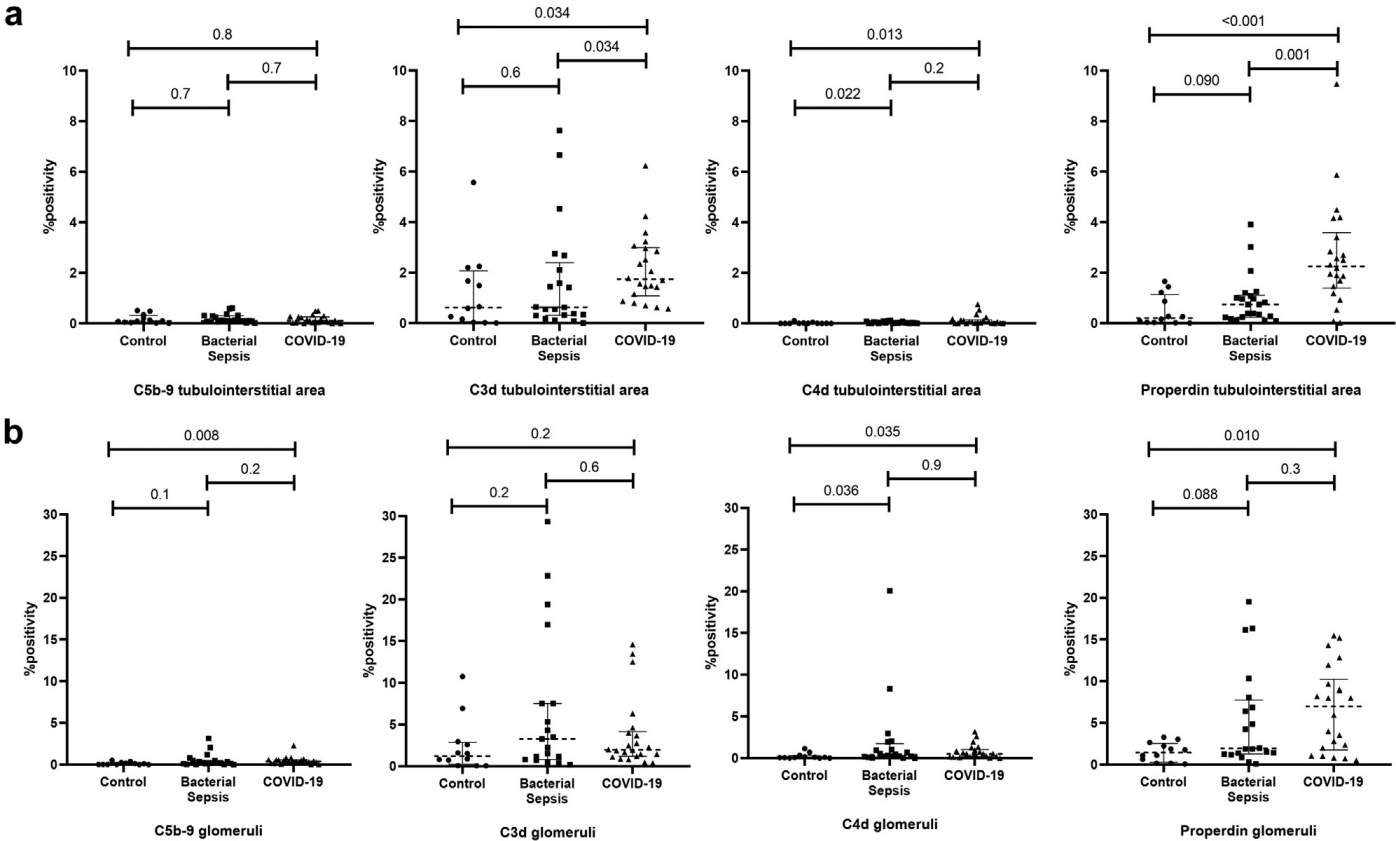
Comparison of the complement factors positivity between controls, patients with bacterial sepsis, and patients with COVID-19. (a) In the tubulointerstitial area. (b) In the glomeruli. Mann-Whitney U test was used to test for differences between groups, and the *P*-value was corrected for multiple comparisons using the false discovery rate method. The correction was performed separately for each complement staining. *P* < 0.05 was considered significant.

**Figure 2:**
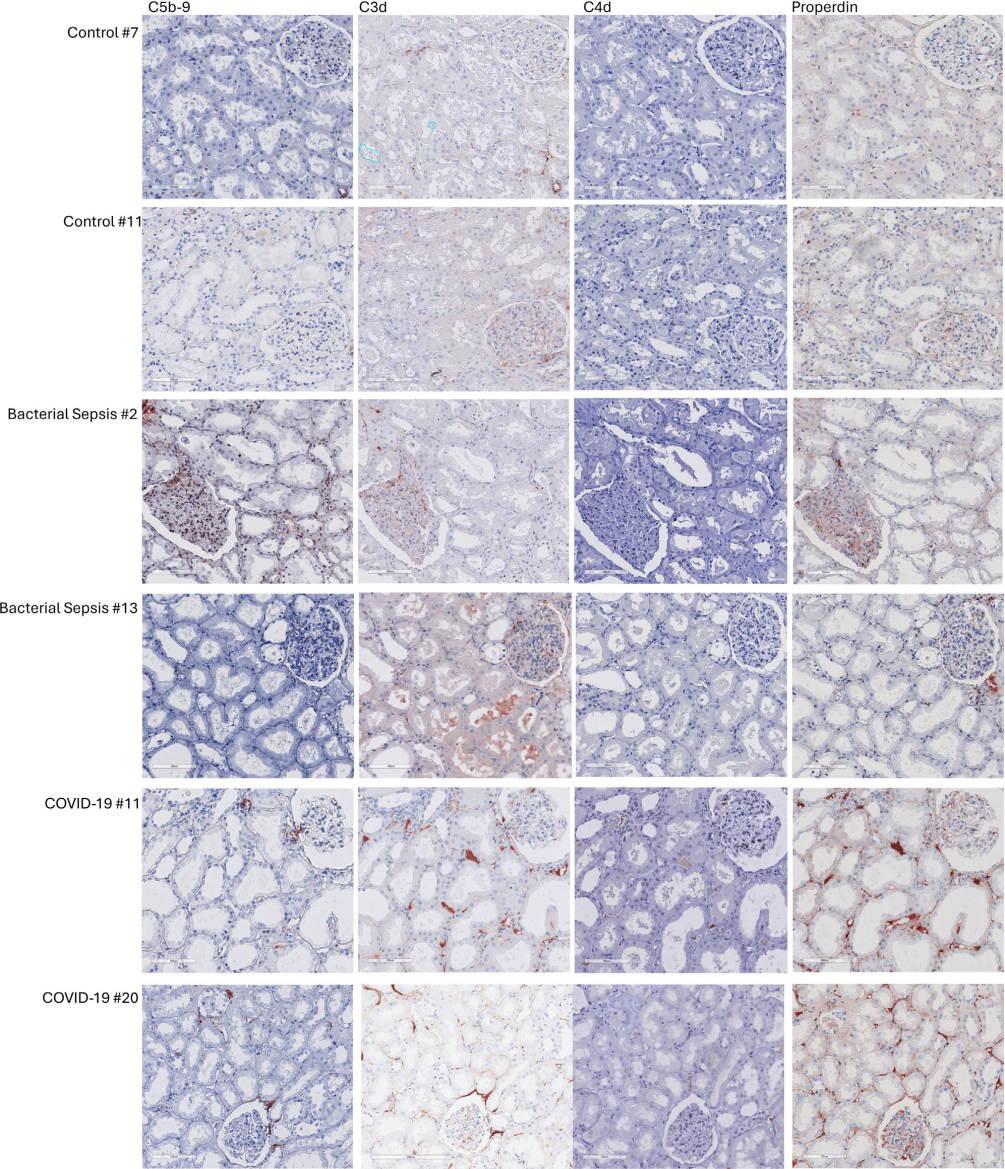
Representative figures of immunohistochemical staining for complement factors in the kidney biopsy in controls, patients with bacterial sepsis, and patients with COVID-19.

In the tubulointerstitial area, C5b-9 positivity was low and similar across groups. The C5b-9 deposition was observed in the tubular basement membranes and not in the peritubular capillaries. In contrast, C3d positivity was significantly higher in the COVID-19 group (1.74 [0.57%–6.23%]) than in control (0.62% [0.01%–5.57%], *P* = 0.034) or bacterial sepsis group (0.63% [0.01%–7.62%], *P* = 0.034). C3d was mainly deposited in peritubular capillaries rather than in the tubular basement membrane. We also found that properdin positivity in the COVID-19 group (2.25 [0.02%–9.47%]) was significantly higher than that in control (0.21% [0.01%–1.66%], *P* < 0.001) or bacterial sepsis group (0.74% [0.09%–3.91%], *P* = 0.001) (Figure 1a). Properdin was also mainly deposited in the peritubular capillaries (Figure 2). If all peritubular capillaries were to stain positive in 1 tubulointerstitial area without any tubular basement staining, the positivity was 3.65%. In addition to properdin, we also evaluated C4d deposition. We found that C4d positivity in general was low; however, in both the bacterial sepsis and COVID-19 groups, it was still significantly higher than that in the control group. Nonetheless, C4d positivity was similar between patients with bacterial sepsis and those with COVID-19 (Figure 1a). The C4d was also mainly deposited in the peritubular capillaries (Figure 2). We did not find significant differences in complement positivity between those with and without peritubular capillary thrombi, between different ATN extensiveness, or between those who were and those who were not clinically diagnosed with AKI in the COVID-19 group (Supplementary Table S5).

Since complement C3d deposition has been shown to be upregulated in the tubular basement membranes of kidneys with ATN in general,^30^ we further compared the C3d deposition in the tubulointerstitial area between ATN control and COVID-19. Unlike the COVID-19 group, the C3d deposition in the ATN control is only present in the tubular basement membranes. There was no C3d deposition in the peritubular capillaries. The C3d staining of each kidney biopsy in the ATN control group is presented in Supplementary Figure S2.

In the glomeruli, C5b-9 positivity was low, similar to that in the peritubular area. Although the C5b-9 positivity was significantly higher in the COVID-19 group than in the control group, there was no difference between COVID-19 and the bacterial sepsis group. Likewise, both properdin and C4d positivity in the glomeruli were significantly higher in COVID-19 than in the control group, but no significant difference was found when comparing COVID-19 with the bacterial sepsis groups. As for the C3d positivity, no significant differences were observed among the 3 groups (Figure 1b). Both C5b-9 and C4d were mostly deposited in the mesangium. As for C3d and properdin, while both were mainly deposited in the mesangial area, glomerular capillary wall deposition was also observed.

## Discussion

This study aimed to compare complement activation in compartments of the kidneys of patients with COVID-19 and those with bacterial sepsis, and to determine the activation pathway. We found that C3d positivity in the tubulointerstitial area, but not in the glomerular area, was significantly higher in the COVID-19 group than in the bacterial sepsis and control groups. The deposition was primarily in the peritubular capillaries rather than in the tubular basement membrane. Regarding the activation pathway, we found that properdin positivity in the tubulointerstitial area was significantly higher in the COVID-19 group, and similar to the C3d, the deposition was primarily in the peritubular capillaries rather than in the tubular basement membranes. In contrast, minor C4d positivity was similar between groups. This indicates that the complement system is markedly activated in the peritubular capillaries of patients with COVID-19, mainly via the alternative pathway.

Similar to our findings, previous studies reported that C3d positivity in the tubulointerstitium, but not in the glomeruli, was significantly higher in COVID-19 kidneys than in control kidneys.^9^, ^10^, ^11^ In addition, we found that tubulointerstitial C3d positivity in the COVID-19 group was significantly higher than that in the bacterial sepsis group, whereas tubulointerstitial C3d positivity in the bacterial sepsis group was similar to that in the control group. Although several previous human studies have evaluated the role of complement in sepsis-induced AKI,^23^^,^^30^ we could not identify studies on the role of complement in sepsis-induced AKI at the human tissue level. Thus, future studies are required to confirm our findings.

In contrast to C3d, we found no differences in the tubulointerstitial C5b-9 positivity across different groups, whereas previous studies reported more C5b-9 deposition in the tubulointerstitial area of the COVID-19 biopsies compared with controls.^8^^,^^10^, ^11^, ^12^ This discrepancy might be explained by the differences in the C5b-9 antibody used for staining. The antibody used in our study (clone A239) strongly binds only to the complete C5b-9 complex, whereas the antibodies used in previous studies (clone aE11 and M0777) strongly bind to both complete and incomplete C5b-9 (i.e., C5b-6, C5b-7, or C5b-8).^37^ Therefore, it is rational to expect that more C5b-9 can be detected using this antibody. However, detecting the incomplete C5b-9 complex is less significant because the cytolytic effect is markedly stronger in the complete C5b-9 complex.^38^

The complement system is tightly regulated by soluble and membrane-bound complement regulatory proteins (CRPs) to avoid excessive complement activation. In the kidney, the expression of 3 membrane-bound CRPs in each segment was evaluated in detail.^39^ One of the evaluated CRPs, CD59, regulates complement at the C5b-9 level by preventing the insertion of C9 into the C5b-8 complex.^40^ CRP is strongly expressed in the peritubular capillaries and, to a lesser extent, in the tubular basement membrane.^39^ This might explain the low and similar C5b-9 positivity in the tubulointerstitial area across different biopsy groups, despite differences in C3d positivity. This phenomenon has been previously reported in the biopsy of kidney transplant recipients with antibody-mediated rejection (ABMR).^32^ In that study, C3d deposition in the peritubular capillary was found to be significantly higher in antibody-mediated rejection than in non–antibody-mediated rejection, whereas C5b-9 deposition was similar.^32^

Between COVID-19 and bacterial sepsis, we found histopathological differences in the presence of peritubular capillary thrombi. However, we did not find differences in the positivity of any complement factors between patients with and without peritubular capillary thrombi in the COVID-19 group. This might be explained by the fact that only 4 of 22 biopsies were without peritubular capillary thrombi, which underpowered the comparative analysis. To overcome this, in a separate analysis, we pooled the sepsis and COVID-19 groups and found significantly higher tubulointerstitial properdin deposition in thrombi-positive biopsies (2.41 [1.39–3.60] vs. 0.87 [0.27–1.36], *P* = 0.003). This suggests that peritubular thrombosis and properdin deposition are interrelated events; however, we do not have more evidence for this suggestion and future animal studies might discover the underlying mechanism.

Of the patients with COVID-19, 68% who were clinically diagnosed with AKI. However, all kidney biopsies from the COVID-19 group revealed structural tubular damage. Furthermore, there was no difference in complement staining positivity between patients who were clinically diagnosed with AKI and those who were not. Kidneys have a reserve capacity for glomerular filtration that can be used when filtration demand increases. Injury to the kidney does not lead to an increase in serum creatinine if the reserve capacity is sufficient.^41^ This may explain why kidney failure was not clinically detected in all these patients despite having tubular injury.

The currently available evidence shows contradictory findings about the complement activation pathway. Two studies concluded that the complement system was activated via the classical and alternative pathways,^9^^,^^12^ another study indicated that complement activation was via the lectin and alternative pathways,^10^ and the other study reported that complement activation was exclusively via the lectin pathway.^11^ In this study, we found that both C4d and properdin positivity were significantly higher in the COVID-19 group than in the control group, although the positivity for C4d was minor (median positivity 13× weaker) compared with that for properdin. Moreover, the deposition of both C4d and properdin was mainly observed in peritubular capillaries. However, when looking at the bacterial sepsis group, we found that C4d but not properdin positivity was significantly higher compared with the control group. In addition, there was no difference in tubulointerstitial C3d positivity between the bacterial sepsis and control groups. Altogether, this suggests that the alternative pathway plays a significant role in complement activation in the tubulointerstitial area of the kidneys of patients with COVID-19. Furthermore, contrary to the suggestion that COVID-19 AKI has a pathophysiology similar to that of bacterial sepsis,^17^, ^18^, ^19^ we showed that there is a distinct difference between these 2 types of AKI, at least regarding complement pathway activation.

In the glomerular area, both the bacterial sepsis and the COVID-19 groups had higher properdin and C4d deposition than the control group; albeit the positivity in the C4d was more subtle than the properdin. However, the C3d positivity in the glomerular area was comparable with either bacterial sepsis or the control group, indicating that the complement system is not activated and causing injury to the glomeruli. This is to be expected, because histopathological changes in COVID-19 kidney biopsy more commonly occur in the tubulointerstitial area rather than in the glomerular area.^5^^,^^16^ In line with this, we found that only 1 patient in each group had histopathological changes in the glomerular area. One possible explanation for complement activation is that the glomerular area has better protection from complement activation because of higher CRP expression.^39^ It is worth noting that the percentage of complement staining positivity in general appears to be higher in the glomerular area than in the tubulointerstitial area because the complement staining in the tubulointerstitial area is predominantly from the peritubular capillaries, and peritubular capillaries cover only a small proportion of the total tubulointerstitial area.

Next to the presence of complement deposition in the peritubular capillaries exclusively in the COVID-19 group and the significantly higher complement deposition in the COVID-19 than in the bacterial sepsis group in the tubulointerstitial area, the COVID-19 group had significantly more extensive ATN than bacterial sepsis. Previously, Thurman *et al.*^30^ found that there was more C3d deposition in the tubular basement membranes of patients with ATN than in the normal controls, whereas C4d was not detectable. Thus, one might argue that the higher complement deposition in the tubulointerstitial area is because of the ATN extensiveness. However, we found that there was no C3d deposition in the peritubular capillaries of the ATN control. Similarly, Thurman *et al.*^30^ did not report any findings regarding C3d deposition in the peritubular capillaries in the kidneys with ATN. Thus, the C3d positivity difference between ATN control and COVID-19 primarily lies in the C3d positivity in the peritubular capillaries, suggesting a role of peritubular complement activation in COVID-19-related kidney pathophysiology.

SARS-CoV-2 RNA was not detected in the kidneys of patients with COVID-19 in our cohort. Previous studies have reported contradictory findings, with some reporting that SARS-CoV-2 was detected in the kidney, whereas others have reported the contrary. The biopsy timing and methods used are of critical importance for detection.^42^ In the current study, because we examined the final stage of the disease, viral tropism might have been cleared from the kidneys. In addition, we only evaluated E-gen and N-gen, whereas spike protein deposition in the kidney tubules has been observed and is thought to induce tubular injury.^8^

Thrombosis is a clinical manifestation of COVID-19-associated coagulopathy, and the complement system plays a pivotal role in this process.^43^^,^^44^ Upon binding to the angiotensin-converting enzyme-2 receptor on the endothelial cell, the SARS-CoV-2 spike protein activates the complement system.^45^^,^^46^ This activation subsequently recruits neutrophils and induces neutrophil extracellular traps via anaphylatoxins C3a and C5a.^47^^,^^48^ The release of neutrophil extracellular traps induces endothelial dysfunction, triggers a proinflammatory immune response, activates extrinsic coagulation cascade clotting by releasing tissue factor, and enhances platelet adhesion.^49^ Simultaneously, C5a promotes platelet adhesion and aggregation by stimulating exocytosis of von Willebrand factor multimers and P-selectin from endothelial cells.^50^ In addition, complement is involved in the coagulation cascade by stimulating the release of tissue factor from neutrophil extracellular traps.^51^ The cumulative result of these events is the development of microvascular and macrovascular thrombosis, which impairs kidney blood flow, leading to tubular injury and eventually kidney failure. From 3 possible activation pathways, a previous *in vivo* study has shown that the SARS-CoV-2 spike protein may activate the complement system via the alternative pathway by interfering with the factor H function.^46^ Another *in vivo* study showed that SARS-CoV-2 might induce alternative pathway complement activation via JAK-STAT1 signaling.^52^ Altogether, this is in line with our findings that complement activation in COVID-19 mainly occurs via an alternative pathway.

The properdin deposition in the tubulointerstitial area was higher in COVID-19 biopsy with less extensive ATN than in COVID-19 biopsy with more extensive ATN, although the difference was not statistically significant. Next to serving as an activator of the alternative pathway, previous studies have shown that properdin may bind to the apoptotic or necrotic cells and facilitate their uptake by phagocytes.^53^, ^54^, ^55^ Because the removal of apoptotic or necrotic cells is important to avoid excessive inflammatory and immune reactions, it then can be speculated that properdin opsonizes damaged tubular cells to be phagocytosed as a meaningful repair response upon injury.^55^ However, the current study setting was not suitable to study the reparative role of properdin, because all of the patients with COVID-19 included in the study died.

Properdin is mainly produced by myeloid cells, including monocytes, macrophages, dendritic cells, and neutrophils.^56^ However, only 3 out of 22 kidney biopsies in the COVID-19 groups had interstitial inflammation, whereas almost all of those biopsies had properdin deposition in the tubulointerstitial area. Therefore, it is unlikely that the properdin production originated from the myeloid cells that infiltrate the tubulointerstitial area. Another possibility for the source of properdin is local production by the tubular epithelial cells. An early study using sieving fractions of human kidney tissue found that properdin expression was detected in the tubular fraction; however, its expression is far weaker than in the glomerular fraction.^57^ Furthermore, the study was done in the early nonquantitative polymerase chain reaction era, and there was no confirmation at the protein level, either by Western blotting or immuno stainings. We also looked into the kidney tissue atlas from the Kidney Precision Medicine Project,^58^ and found that the mean expression of properdin by the tubular epithelial cells is low. Based on this, we argue that it is unlikely that tubular epithelial cells are the source of properdin production. Because we largely rule out potential local sources of properdin production in the kidney, this leaves the likely possibility of docking of systemic properdin on the peritubular endothelium.

Considering that the complement system is involved in the pathogenesis of COVID-19 AKI, targeting the complement system might be a potential therapeutic approach. Currently, only inhibition at the C5 level has been approved for use in the clinical setting for treating patients with COVID-19.^59^ A previous systematic review and meta-analysis showed that although inhibition of C5 levels significantly reduced the risk of mortality, the risk of developing AKI was similar between the treated and control groups.^60^ Thus, blocking upstream complement activation may be a better approach to prevent the development of AKI and reduce the risk of mortality. Currently, there are other ongoing trials that target upstream complement activation^61^; however, whether any of these drugs can reduce the risk of developing AKI remains to be seen.

The strength of this study is that it is the first study to evaluate properdin deposition and compare complement activation in the kidneys of patients with COVID-19 and those with bacterial sepsis, adding novelty to the AKI research field. However, there are several important limitations to our study. First, we only studied biopsies from deceased patients, which limits our results to the sickest patient group. Second, the biopsy was performed after a prolonged variable process of illness and death; thus, the initial pathological process could not be captured. Third, we used paraffin-embedded kidney biopsy material for all complement staining, and this material is prone to the presence of serum trapping that might give false positive results, although we tried our very best by adding a number of different control biopsies to avoid this possibility. To overcome this, frozen tissue should be used to confirm the findings. Unfortunately, we did not have frozen tissue available. Fourth, von Willebrand factor is known to be involved in the pathogenesis of complement-mediated thrombosis by serving as a platform for alternative pathway complement activation on the endothelial cell surface.^62^ However, we were not able to perform von Willebrand factor staining on the paraffin-embedded kidney biopsy material to see if the thrombi-positive peritubular capillaries were rich in von Willebrand factor. Lastly, the histopathological scoring could not be blinded, which may have introduced observer bias.

In conclusion, we found marked tubulointerstitial complement deposition in the kidneys of patients with COVID-19, especially in the peritubular capillaries, with activation predominantly via the alternative pathway. These findings further support the involvement of the complement system in COVID-19 pathogenesis, particularly in the development of peritubular microvascular thrombosis. Thus, inhibition of the alternative pathway may be a possible treatment option for COVID-19-related AKI.

## Disclosure

All the authors declared no competing interests.

## Supplementary Material

Supplementary File (PDF) **Figure S1.** An example of the percentage of positivity quantification. **Figure S2.** Complement C3d staining in acute tubular necrosis control. **Table S1.** Summary of previous studies that evaluate complement activation pathways in the kidney of patients with COVID-19. **Table S2.** Scoring methods for the histopathological evaluation of kidney biopsies. **Table S3.** Detailed complement staining protocol. **Table S4.** Individual histopathology score from bacterial sepsis, COVID-19, and acute tubular necrosis control patients. **Table S5.** Comparison of complement factors positivity in the tubulointerstitial areas between patients with COVID-19 with and without thrombi in the peritubular capillary, with different acute tubular necrosis extensiveness, and with and without acute kidney injury.

## Footnotes

Supplementary File (PDF)
**Figure S1.** An example of the percentage of positivity quantification. **Figure S2.** Complement C3d staining in acute tubular necrosis control. **Table S1.** Summary of previous studies that evaluate complement activation pathways in the kidney of patients with COVID-19. **Table S2.** Scoring methods for the histopathological evaluation of kidney biopsies. **Table S3.** Detailed complement staining protocol. **Table S4.** Individual histopathology score from bacterial sepsis, COVID-19, and acute tubular necrosis control patients. **Table S5.** Comparison of complement factors positivity in the tubulointerstitial areas between patients with COVID-19 with and without thrombi in the peritubular capillary, with different acute tubular necrosis extensiveness, and with and without acute kidney injury.
