# Supplementary material for: Complement Activation is More Pronounced in the Kidneys of Critically Ill Patients With COVID-19 Than in Those With Bacterial Sepsis
Source: Kidney Int Rep. 2025 Apr 21;10(7):2284–95. doi: 10.1016/j.ekir.2025.04.027 (PMC12266222; doi:10.1016/j.ekir.2025.04.027)
Supplement: Supplementary File (PDF) — Figure S1. An example of the percentage of positivity quantification. Figure S2. Complement C3d staining in acute tubular necrosis control. Table S1. Summary of previous studies that evaluate complement activation pathways in the kidney of patients with COVID-19. Table S2. Scoring methods for the histopathological evaluation of kidney biopsies. Table S3. Detailed complement staining protocol. Table S4. Individual histopathology score from bacterial sepsis, COVID-19, and acute tubular necrosis control patients. Table S5. Comparison of complement factors positivity in the tubulointerstitial areas between patients with COVID-19 with and without thrombi in the peritubular capillary, with different acute tubular necrosis extensiveness, and with and without acute kidney injury. [file mmc1.pdf]

Complement Activation is More Pronounced in the Kidneys of Critically Ill Patients With COVID-19 Than in Those With Bacterial Sepsis

SUPPLEMENTAL MATERIAL

Table of contents

**Supplementary Figure 1.** An example of the percentage of positivity quantification.	Page 2
**Supplementary Figure 2.** Complement C3d staining in acute tubular necrosis control.	Page 3
**Supplementary Table 1.** Summary of previous studies that evaluate complement activation pathways in the kidney of COVID-19 patients.	Page 4
**Supplementary Table 2.** Scoring methods for the histopathological evaluation of kidney biopsies	Page 6
**Supplementary Table 3.** Detailed complement staining protocol.	Page 7
**Supplementary Table 4.** Individual histopathology score from bacterial sepsis, COVID-19, and acute tubular necrosis control patients.	Page 8
**Supplementary Table 5.** Comparison of complement factors positivity in the tubulointerstitial areas between COVID-19 patients with and without thrombi in the peritubular capillary, with different acute tubular necrosis extensiveness, and with and without acute kidney injury.	Page 11

Supplementary Figure 1. An example of the percentage of positivity quantification.

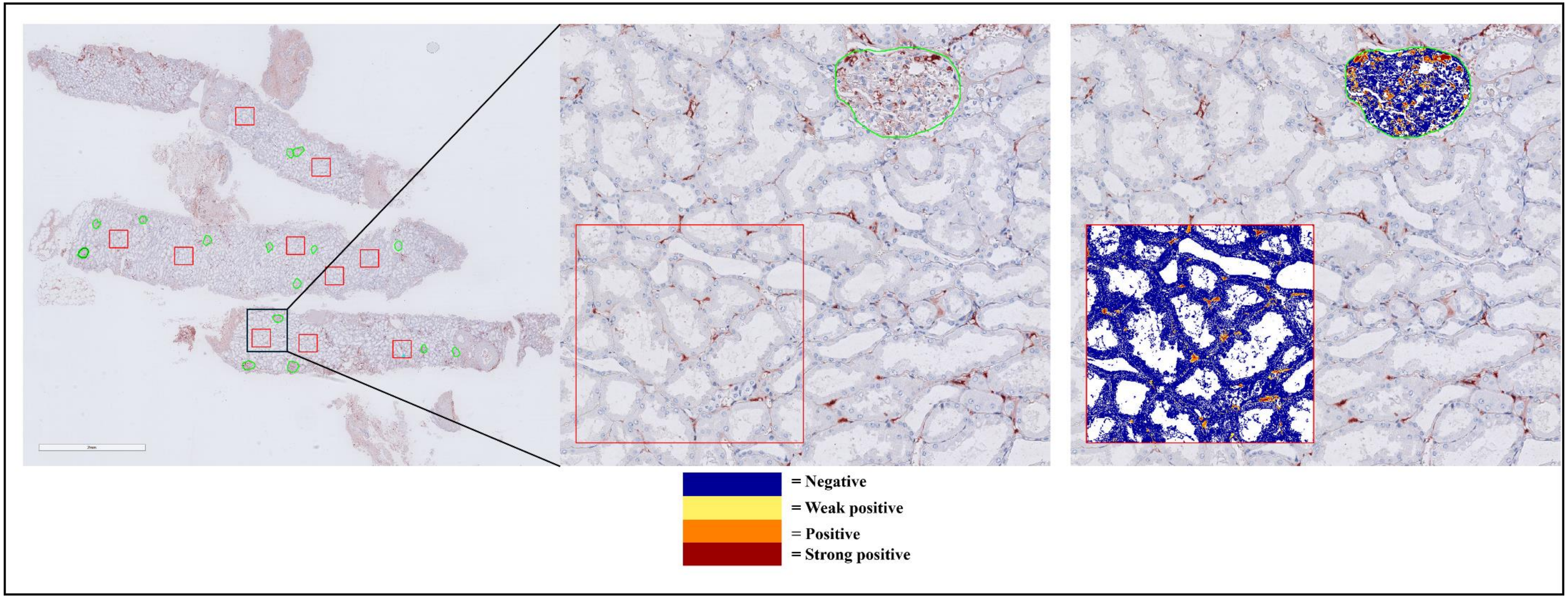

This biopsy of COVID-19 #18 was stained for complement factor C3d. Fifteen glomeruli and 10 tubulointerstitial areas were selected for the quantification. The percentage of positivity was defined as the sum of positive and strong positive pixels multiplied by 100 and divided by the total pixel in the selected area. The percentage of positivity in this example was 15.96% for the glomerulus and 3.60% for the peritubular area. Later, the median percentage of positivity from all glomeruli (or peritubular area) was used in the analysis.

Supplementary Figure 2. Complement C3d staining in acute tubular necrosis control.

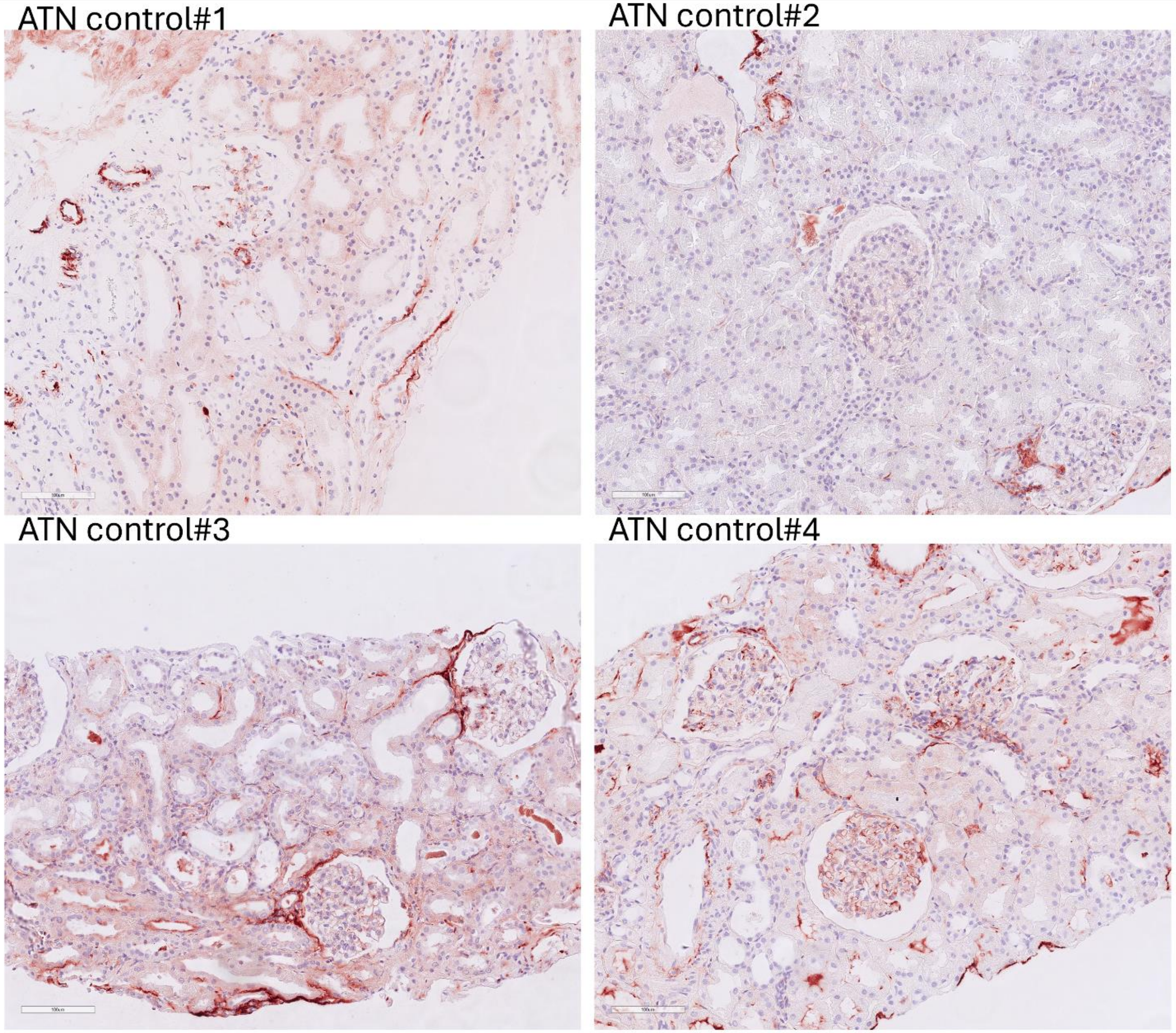

Complement C3d staining of each kidney biopsy in the acute tubular necrosis (ATN) control group.

Supplementary Table 1. Summary of previous studies that evaluate complement activation pathways in the kidney of COVID-19 patients.

Authors & year	Country	N	Biopsy type	Evaluated complement factors	Conclusion regarding the complement activation pathway	Treatment setting
Jamaly et al (2021)[9]	USA	3	Postmortem	C1q, factor H, C3, C5b-9	Via classical and alternative pathways	Intensive care unit (3)
Pfister et al (2021)[10]	Germany	9	Diagnostic and postmortem	C1q, MASP-2, C4d, C3c, C3d, C5b-9	Via lectin and alternative pathways	No information
Macor et al (2021)[12]	Italy	12	Postmortem	C1q, C4, C3, C5b-9	Via classical and alternative pathways	Intensive care unit (2)<br>Ward (10)
Niederreiter et al (2022)[11]	Germany	38	Postmortem	C1q, MASP-2, factor D, C3c, C3d, C5b-9	Via lectin pathway	No information

The search was conducted on 18 September 2024 and updated on 21 March 2025 in three different databases using the following search terms:

1. PubMed

   ("COVID-19"[MeSH Terms] OR "SARS-CoV-2"[MeSH Terms] OR "COVID-19"[Title/Abstract] OR "SARS-CoV-2"[Title/Abstract]) AND ("Complement Activation"[MeSH Terms] OR "Complement System Proteins"[MeSH Terms] OR "complement*"[Title/Abstract]) AND ("autopsy"[MeSH Terms] OR "autop*"[Title/Abstract] OR "biopsy"[MeSH Terms] OR "biop*"[Title/Abstract] OR "tissue*"[Title/Abstract]) AND ("Kidney"[MeSH Terms] OR "kidney*"[Title/Abstract] OR "renal"[Title/Abstract])

2. EMBASE

('covid-19'/exp OR 'sars-cov-2'/exp OR covid-19:ab,ti OR sars-cov-2:ab,ti) AND ('complement activation'/exp OR 'complement system'/exp OR complement*:ab,ti) AND ('autopsy'/exp OR 'biopsy'/exp OR autop*:ab,ti OR biop*:ab,ti OR tissue*:ab,ti) AND ('kidney'/exp OR 'renal function'/exp OR kidney*:ab,ti OR renal:ab,ti) AND ('article'/it OR 'article in press'/it OR 'letter'/it)

3. Web of Science

TS=("COVID-19" OR "SARS-CoV-2") AND TS=("Complement Activation" OR "Complement System Proteins" OR complement*) AND TS=(autop* OR biop* OR tissue*) AND TS=(kidney* OR renal)

Supplementary Table 2. Scoring methods for the histopathological evaluation of kidney biopsies.

**GLOMERULUS**	
**Glomerulitis**	0: No, < 10 leukocytes in glomerular capillaries
	1: Yes, ≥ 10 leukocytes in glomerular capillaries
**Thrombi glomerular**	0: No
	1: Yes
**Increase of mesangial matrix**	0: no mesangial matrix increase
	1: ≤ 25% of non-sclerotic glomeruli
	2: 26-50% of non-sclerotic glomeruli
	3: > 50% of non-sclerotic glomeruli.

**Tobulointerstitial**	
**Acute Tubular Necrosis Morphology**	0: absent
	1: tubulointerstitial edema, dilatation, thinning tubular epithelia
	2: apoptotic tubular epithelial cell, casts, signs of regeneration
	3: signs of extensive tubular loss
**Acute Tubular Necrosis Extensiveness**	0: absent
	1: only rare tubules with signs of ATN
	2: small discontinuous groups of tubules with signs of ATN
	3: easily found larger groups of tubules with signs of ATN
	4: extensive areas of tubules with signs of ATN
**Tubulitis**	0: no mononuclear cells in tubules
	1: 1-4 mononuclear cells/tubular cross-section
	2: 5-10 mononuclear cells/tubular cross-section
	3: >10 mononuclear cells/tubular cross-section
**Interstitial Inflammation**	0: no or hardly any mononuclear cells in the interstitial parenchyma
	1: 10-25% interstitial parenchyma covered with mononuclear cells
	2: 26-50% interstitial parenchyma covered with mononuclear cells
	3: >50% interstitial parenchyma covered with mononuclear cells.
**Interstitial fibrosis/tubular atrophy**	0: absent
	1: mild (<25% of the total area)
	2: moderate (25-50% of the total area)
	3: severe (>50% of the total area)
**Peritubular capillaritis**	0: <10% capillaries containing inflammatory cells
	1: >10% capillaries containing 3-4 luminal cells
	2: >10% capillaries containing 5-10 luminal cells
	3: >10% capillaries containing >10 luminal cells
**Thrombi peritubular capillaries**	0: No
	1: Yes

Supplementary Table 3. Detailed complement staining protocol.

Antigen	Antigen retrieval	Blocking step	Antibody	Conjugate 1	Conjugate 2	Substrate	Control sample
C5b-9	0,03% protease in PBS for 30 minutes at room temperature	0.3% H_2O_2 in PBS for 30 minutes at room temperature	Monoclonal mouse anti-human (Quidel, A239), 1:10,000 dilution in 1% BSA in PBS, overnight at room temperature	polyclonal rabbit anti-mouse HRP (DAKO, P0260), 1:100 dilution in 1% BSA in PBS + 1% normal human antibody serum, for 30 minutes at room temperature	polyclonal goat anti-rabbit HRP (DAKO, P0448), 1:100 dilution in 1% BSA in PBS + 1% normal human antibody serum for 30 minutes at room temperature	Ready-to-use AEC substrate kit (ABCAM ab64252, undiluted) at room temperature for 20 minutes	Pre-transplantation biopsy of a deceased donor and kidney biopsy of a patient with primary membranous nephropathy
C3d	0,4% pepsin in demi-water for 30 minutes at room temperature		polyclonal rabbit anti-human (DAKO, A0063), 1:2,500 dilution in 1% BSA in PBS, overnight at room temperature	polyclonal goat anti-rabbit HRP (DAKO, P0448), 1:100 dilution in 1% BSA in PBS, for 30 minutes at room temperature	polyclonal rabbit anti-goat HRP (DAKO, P0449), 1:100 dilution in 1% BSA in PBS, for 30 minutes at room temperature		
Properdin	0,03% protease in PBS for 30 minutes at room temperature		polyclonal rabbit anti-human (Laboratory of Nephrology, Leiden, the Netherlands), 1:400 dilution in 1% BSA in PBS, overnight at room temperature				

Supplementary Table 4. Individual histopathology score from bacterial sepsis, COVID-19, and acute tubular necrosis control patients.

**Bacterial sepsis**	**1**	**2**	**3**	**4**	**5**	**6**	**7**	**8**	**9**	**10**	**11**	**12**	**13**	**14**	**15**	**16**	**17**	**18**	**19**	**20**	**21**	**22**
**RIFLE AKI stage**	I	I	F	F	F	I	I	F	F	F	F	F	F	F	F	I	I	F	I	F	F	I
**Renal replacement therapy**	N	N	Y	N	Y	N	N	Y	N	N	Y	Y	Y	Y	Y	N	N	Y	N	Y	Y	N
**Glomeruli**																						
Glomerulitis	0	0	0	0	0	0	0	0	0	0	0	0	0	0	0	0	0	0	0	0	0	0
Thrombi glomerular	0	0	0	0	0	0	0	0	0	0	0	0	0	0	1	0	0	0	0	0	0	0
Increase of mesangial matrix	0	0	0	0	0	0	0	0	0	0	0	0	0	0	0	0	0	0	0	0	0	0
**Tubulointerstitial**																						
Acute tubular necrosis morphology	2	2	2	2	0	2	2	2	2	2	2	0	2	2	3	2	2	0	1	1	1	2
Acute tubular necrosis extensiveness	1	1	1	1	0	1	1	1	1	1	1	0	1	1	3	2	2	0	1	1	1	3
Tubulitis	0	0	0	0	0	0	0	0	0	0	0	0	0	0	0	0	0	0	0	0	0	0
Interstitial inflammation	0	0	0	0	0	0	0	1	0	0	0	1	0	0	0	0	0	0	0	0	0	0
Interstitial fibrosis/tubular atrophy	1	0	1	0	0	0	1	0	1	0	0	1	0	0	1	0	0	0	0	1	0	0
Peritubular capillaritits	0	0	0	0	0	0	0	0	0	0	0	0	0	0	0	0	0	0	0	0	0	0
Thrombi peritubular capillaries	0	0	0	0	0	0	0	0	0	0	0	0	0	0	0	0	0	0	0	0	0	0

COVID-19	1	2	3	4	5	6	7	8	9	10	11	12	13	14	15	16	17	18	19	20	21	22
**KDIGO AKI stage**	0	3	3	3	3	3	3	3	3	3	3	3	3	3	0	3	0	0	0	0	0	3
**Renal replacement therapy**	N	Y	Y	Y	N	Y	N	N	N	Y	Y	N	N	Y	N	N	N	N	N	N	N	Y
**Glomeruli**																						
Glomerulitis	0	0	0	0	0	0	0	0	0	0	0	0	0	0	0	0	0	0	0	0	0	0
Thrombi glomerular	0	0	0	1	0	0	0	0	0	0	0	0	0	0	0	0	0	0	0	0	0	0
Increase of mesangial matrix	0	0	0	0	0	0	0	0	0	0	0	0	0	0	0	0	0	0	0	0	0	0
**Tubulointerstitial**																						
Acute tubular necrosis morphology	2	2	2	1	2	2	1	1	1	2	2	2	2	2	1	1	2	2	1	2	1	2
Acute tubular necrosis extensiveness	2	3	3	2	2	2	2	2	2	3	3	2	2	3	2	2	2	3	3	3	2	3
Tubulitis	0	0	0	0	0	1	0	0	0	1	0	0	0	0	0	0	0	0	0	0	0	0
Interstitial inflammation	0	0	0	0	0	1	0	0	0	1	0	0	0	0	0	0	0	0	0	0	0	1
Interstitial fibrosis/tubular atrophy	0	1	0	0	0	0	0	1	1	0	0	0	0	0	0	0	1	0	0	0	0	1
Peritubular capillaritits	0	0	1	0	0	0	0	0	0	0	0	0	0	0	0	0	0	0	0	0	0	0
Thrombi peritubular capillaries	0	1	1	1	1	1	0	1	1	1	1	1	1	1	1	1	0	1	1	1	1	0

ATN control	1	2	3	4
**Glomeruli**				
Glomerulitis	0	0	0	0
Thrombi glomerular	0	0	0	0
Increase of mesangial matrix	0	0	0	0
**Tubulointerstitial**				
Acute tubular necrosis morphology	3	1	3	1
Acute tubular necrosis extensiveness	3	2	3	2
Tubulitis	0	0	0	0
Interstitial inflammation	1	1	1	1
Interstitial fibrosis/tubular atrophy	0	0	0	0
Peritubular capillaritits	0	0	0	0
Thrombi peritubular capillaries	0	0	0	0

*The detailed explanation of the histopathology score is presented in Supplementary Table 2.

Supplementary Table 5. Comparison of complement factors positivity in the tubulointerstitial areas between COVID-19 patients with and without thrombi in the peritubular capillary, with different acute tubular necrosis extensiveness, and with and without acute kidney injury.

**%positivity**	**Peritubular capillary thrombi**			**Acute tubular necrosis extensiveness**			**KDIGO AKI stage**		
	Absent<br>N = 4	Present<br>N = 18	p-value	2<br>N = 13	3<br>N = 9	P-value	0<br>N = 7	3<br>N = 15	p-value
C5b-9	0.25<br>[0.11 – 0.34]	0.06<br>[0.00 – 0.50]	0.096	0.11<br>[0.00 – 0.50]	0.12<br>[0.00 – 0.49]	0.9	0.06<br>[0.03 – 0.50]	0.12<br>[0.00 – 0.49]	0.9
C3d	2.69<br>[1.70 – 3.58]	1.52<br>[0.57 – 6.23]	0.2	1.78<br>[0.62 – 4.23]	1.56<br>[0.57 – 6.23]	0.9	2.05<br>[0.57 – 6.23]	1.70<br>[0.62 – 3.23]	0.5
C4d	0.08<br>[0.01 – 0.14]	0.06<br>[0.00 – 0.76]	1.0	0.05<br>[0.00 – 0.76]	0.14<br>[0.00 – 0.54]	0.3	0.12<br>[0.02 – 0.76]	0.05<br>[0.00 – 0.54]	0.1
Properdin	1.78<br>[0.92 – 4.16]	2.41<br>[0.02 – 9.47]	0.4	2.50<br>[0.02 – 9.47]	1.46<br>[0.08 – 5.87]	0.1	2.50<br>[1.19 – 4.49]	2.19<br>[0.02 – 9.47]	0.5

KDIGO AKI, Kidney Disease: Improving Global Outcomes Acute kidney injury.
